# Relationship Between Low Education and Poor Periodontal Status among Mexican Adults Aged ≥50 Years

**DOI:** 10.3290/j.ohpd.b5779170

**Published:** 2024-10-14

**Authors:** Alvaro García Pérez, Jacqueline Adelina Rodríguez Chávez, Kathia Guadalupe Rodríguez González, Juan Carlos Cuevas González, Teresa Villanueva Gutiérrez, Laura Bárbara Velázquez-Olmedo, Alejandra Moreno Altamirano

**Affiliations:** a Professor, Laboratory of Public Health Research, Faculty of Higher Studies Iztacala, National Autonomous University of Mexico (UNAM), State of Mexico, Mexico. Designed the study, contributed to data analysis, wrote and reviewed the manuscript, contributed to discussion.; b Professor, Department of Comprehensive Dental Clinics, University Center for Health Sciences, University of Guadalajara, Guadalajara, Mexico. Reviewed the manuscript, contributed to the discussion.; c Professor, Master and Doctoral Program in Medical, Dental and Health Sciences, Faculty of Dentistry, National Autonomous University of México, Mexico City, Mexico. Reviewed the manuscript, contributed to the discussion.; d Professor, Institute of Biomedical Sciences, Autonomous University of Ciudad Juárez, Ciudad Juárez, Mexico. Reviewed the manuscript, contributed to the discussion.; e Professor, Health Care Department, Metropolitan Autonomous University-Xochimilco, Mexico. Reviewed the manuscript, contributed to the discussion.; f Professor, Faculty of Dentistry, National Autonomous University of Mexico (UNAM), Mexico City, Mexico. Reviewed the manuscript, contributed to the discussion.; g Professor, Department of Public Health, School of Medicine, National Autonomous University of Mexico (UNAM), Mexico City, Mexico. Reviewed the manuscript, contributed to the discussion.

**Keywords:** diabetes, level of education, older adults, periodontal disease, periodontal pockets

## Abstract

**Purpose:**

To examine the association between educational level and the presence of periodontal disease in adults ages ≥ 50 years in Mexico.

**Materials and Methods:**

A cross-sectional study was conducted on 2098 Mexican adults, using data from the annual reports of the Epidemiological Monitoring System for Oral Pathologies from 2019–2022. Data were collected on sociodemographic characteristics such as gender, age, educational level, oral hygiene, and diabetes. Periodontal status was evaluated using the Community Periodontal Index (CPI) and was classified into: CPI = 0 (healthy); CPI = 1 (bleeding on probing); CPI = 2 (calculus); and CPI = 3 or 4 (pocket depth ≥ 4 mm). A multinomial regression model was used to estimate the odds ratio (OR) and the 95% confidence intervals (CI), using periodontal status as the result.

**Results:**

39.9% of subjects presented periodontal pockets of ≥ 4 mm, 20.8% presented calculus, and 12.8% presented bleeding, while only 26.4% were classified as healthy. A low level of education (≤ 9 years) (OR = 4.84; p < 0.001), age ≥ 65 years (OR = 1.33; p = 0.025), poor oral hygiene (OR = 6.86; p < 0.001), smoking (OR = 1.51; p = 0.025), and diabetes (OR = 1.73; p < 0.001) were statistically significantly associated with the presence of periodontal pockets ≥ 4 mm.

**Conclusions:**

A low level of education is associated with worse periodontal status in adults aged 50 years or more. These findings reiterate the importance of implementing effective strategies and the incorporation of interventions for improving the access to and quality of services targeted at aging communities.

Aging is a physiological, dynamic, complex, and individualised process experienced during a person’s lifetime and, in turn, has biological, psychological, and social effects. The biological changes that occur with age produce both diminished bodily functions and diverse physiological changes in the various organs and systems of the body.^10^

Aging has become a topic of global interest for both public policy and healthcare systems, due to the growing healthcare needs of adults and older adults and the demand for services indispensable to these populations. For decades, aging has been associated with a diverse range of chronic conditions^31,32^ and oral diseases that may have a significant impact on health, well-being, and quality of life in adults.^22^ One of these is periodontal disease, which is currently considered a public health problem and is related to systemic diseases, such as diabetes.^23,25^ It presents as a chronic infection that causes the destruction of the supporting tissues of the teeth (gingiva, periodontal ligaments, root cementum, and the alveolar bone) as a result of the gingival inflammation caused by the accumulation of plaque and calculus which, if not treated, progresses into periodontitis.^19^ Research on periodontal disease has mainly investigated the composition of subgingival biofilms at sites of advanced periodontal tissue destruction. However, interest has recently been generated in investigating the role played by specific bacteria, such as *A. actinomycetemcomitans, C. rectus, P. gingivalis, P. intermedia/P. intermedia, T. forsythia* and *T. denticola*, in deeper periodontal pockets.^2,28^

The Global Oral Health Status Report 2022 estimated that oral diseases affect nearly 3.5 billion people at a global level.^42^ The 2019 Global Burden of Disease Study reported 1.1 billion cases of severe/serious periodontitis, while from 1990 to 2019, the prevalence of severe periodontitis increased by 8.4% worldwide.^7^

Poor oral hygiene and smoking are not the only significant risk factors for the occurrence of periodontal disease.^8^ Other factors are associated with this disease, e.g., social inequalities observed in certain populations, lack of access to healthcare services, difficult financial situations, a low level of education, a lack of health-related information and education, and living in a rural area.^20^ Global estimates of direct treatment costs and productivity losses due to periodontitis (including for periodontitis-related tooth loss) reached approximately US$ 186 billion and US$ 142 billion, respectively, in 2019.^24^

Low levels of education continue to be common in aging populations, particularly in low- and middle-income countries.^35^ Mexico is currently experiencing an increase in educational level, wherein the percentage of adults age 50 years or over and with six or more years of education increased from 30.9% in 2000 to 45.2% by 2012.^6^ Various recent studies have reported that a low level of education may be a predictor for the occurence of dementia, functional disability, fragility,^3,5^ and periodontal disease in adults and older adults.^43^ According to the information available in the literature, the physical consequences of severe periodontal disease reduce an individual’s functional capacity to communicate or eat, particularly when the teeth are loose or have been lost, which affects social interaction, general well-being, and quality of life for adults.^37^

It can be said that populations with low education present poor oral health and, as a result, may experience consequences in the functional abilities of their oral cavity. Ascertaining the relationship between educational level and periodontal disease in aging populations in Mexico will assist in generating appropriate strategies and measures for improving oral health-related behaviour and care in adults and older adults, whose demands and oral health needs must be met. Therefore, the present study aimed to examine the association between educational level and the presence of periodontal disease in adults ages ≥50 years in Mexico. The research hypothesis was that a low level of education is related to poor periodontal status in Mexican adults aged ≥50 years.

## MATERIALS AND METHODS

This is a retrospective cross-sectional study. Its research protocol was reviewed and approved by the Ethics Committee of the Iztacala Faculty of Higher Studies at the National Autonomous University of Mexico (CE/FESI/032023/1587), and the study itself was conducted in accordance with the Declaration of Helsinki.

### Data Collection

The periodontal status of the adults participating in the present study was evaluated using data taken from a series of annual reports (2019–2022) by the Sistema de Vigilancia Epidemiológica de Patologías Bucales (SIVEPAB or the Epidemiological Monitoring System for Oral Pathologies), which is administered by the Ministry of Health General Directorate for Epidemiology. Among the responsibilities of SIVEPAB is the collection of data on patients seeking dental care, mainly from “primary care services”, at one of the 442 sentinel units located in the 32 states of Mexico. The present study used non-probability convenience sampling methods. The inclusion criteria for participants in the present study were being a patient 50 years of age or more, of either gender, who did not present missing data in the database. Patients with third molars were excluded.

### Independent Variable: Level of Education

The variable “years of education” was used to compare those adults who had completed ≤ 9 years of formal education with those who had completed more than 9 years, which, in Mexico, corresponds to primary and secondary school combined. The independent variable was dichotomized into ≤ 9 years and > 9 years.

### Dependent Variable: Periodontal Status

The present study used the CPI, which measures the prevalence and severity of periodontal disease, and also indicates the corresponding treatment needs. Moreover, the CPI was used to document probing depth, on a scale of 0 to 4, corresponding to CPI = 0 (healthy), CPI = 1 (bleeding on probing), CPI = 2 (calculus), CPI = 3 (pocket depth of 4 to 5 mm), and CPI = 4 (pocket depth of ≥ 6 mm). A complete examination of the oral cavity was performed. The oral cavity was divided into sextants with the presence of at least two functional teeth. According to the CPI criteria, if no first or second molar was present in a sextant, all the teeth present were examined. All the adult participants were examined by dentists, using a periodontal probe WHO, in a dental chair equipped with a light source. For each participant, the final CPI score corresponded to the most severe score of the CPI readings obtained from the six sextants.^41^

### Covariables

The present study used the following sociodemographic variables, with a model fit for potential confounders: age (in years), categorised into two groups (50–64 years and ≥ 65 years); gender (male/female); smoking, categorised into two groups (never/former smoker and current); diabetes (yes/no); and oral hygiene, evaluated using the Simplified Oral Hygiene Index (OHI-S). The index is used to evaluate plaque and calculus debris on selected tooth surfaces. The vestibular and lingual surfaces of six permanent teeth were examined. Oral hygiene was classified as poor (OHI-S score ≥2) or good (OHI-S score <2).^41^

### Sample Size

The sample size was calculated using the formula for two independent proportions with 80% power, with a 0.17 difference in proportions detected between the two groups, and a bilateral p-value of 0.05. Assuming that 48% of the participants of the population investigated present the factor of interest (CPI 3-4), the present study required a sample size of 204 per group, i.e., a total sample size of 408, assuming equal-sized groups.^9^

### Statistical Analysis

All statistical analyses were carried out using the Stata 15 program (Stata; College Station, TX, USA). Chi-squared tests were used to determine associations among the variables of age, gender, oral hygiene, smoking, level of education, and diabetes for the groups obtained using the CPI. Multinomial regression was used to analyse the association among the independent variables (age, gender, oral hygiene, level of education, smoking, and diabetes) and the dependent variable “periodontal status” (CPI categories), which was expressed as an odds ratio (OR) with 95% confidence intervals (CI). Possible interactions between diabetes and level of education were analysed. The participants were classified into four groups according to their periodontal status: CPI = 0; CPI = 1; CPI = 2; and CPI = 3/4. In all analyses, two-tailed values of p < 0.05 were considered statistically significant.

## RESULTS

### Characteristics of the Study Population

**Fig 1:**
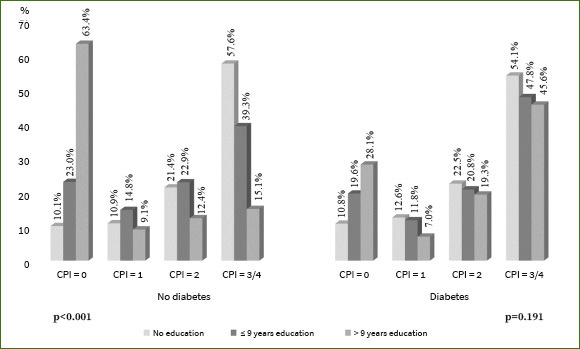
Percent distribution between of the CPI score and the educational level by diabetes among Mexican adults aged ≥ 50 years (n = 2098).

**Fig 2:**
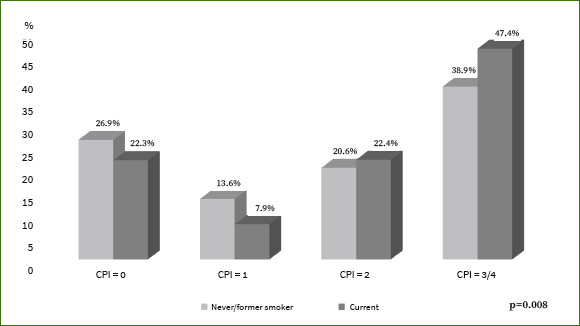
Percent distribution between of the CPI score and smoking status among Mexican adults aged ≥ 50 years (n = 2098).

The present study was conducted on 2098 adults ages ≥ 50 years, with a mean age of 62.0 (±9.4) years, of whom 59.5% were women. In terms of the number of years of education, 83.1% had ≤ 9 years and 16.9% > 9 years. According to the OHI-S, 62.1% of the adults had poor oral hygiene. In terms of the worst finding for periodontal status of the participants, 39.9% presented periodontal pockets ≥ 4 mm, 20.8% had calculus, and 12.8% showed bleeding, while only 26.4% were classified as healthy. The prevalence of diabetes in the participants was 23.4% and was higher in women than in men (27.1% vs 17.8%; p < 0.001). The characteristics of the participants, according to their periodontal status, are shown in Table 1. The male participants presented a higher percentage of periodontal pockets ≥ 4 mm than did female participants (p = 0.025); smokers with poor oral hygiene and diabetes presented the highest percentage of periodontal pockets ≥ 4 mm. Similarly, participants with a low level of education (≤ 9 years) presented a higher number of periodontal pockets ≥ 4 mm than those with >9 years of education (p < 0.001). Figure 1 shows that in participants with diabetes, the percentage of periodontal pockets ≥ 4 mm was similar in the three categories of educational level among participants with diabetes (p = 0.191). The percentage of periodontal pockets ≥ 4 mm was lowest in participants with > 9 years of education and without diabetes (p < 0.001). Figure 2 shows that participants who currently smoke have more periodontal pockets ≥4 mm compared to participants who have never smoked or were former smokers (p = 0.008).

**Table 1:** Distribution of variables by CPI score among Mexican adults aged ≥ 50 years (n = 2098)

	CPI = 0 n = 554 n (%)	CPI = 1 n = 269 n (%)	CPI = 2 n = 437 n (%)	CPI = 3/4 n = 838 n (%)	p-value*
Age (years)					
50–64	389 (70.2)	174 (64.7)	310 (70.9)	471 (56.2)	<0.001
≥ 65	165 (29.8)	95 (35.3)	127 (29.1)	367 (43.8)	
Gender					
Female	221 (39.9)	105 (39.0)	204 (46.7)	319 (38.1)	0.025
Male	333 (60.1)	164 (61.0)	233 (53.3)	519 (61.9)	
Oral hygiene (OHI-S)					
Good hygiene	489 (88.3)	158 (58.7)	248 (56.7)	407 (48.6)	<0.001
Poor hygiene	65 (11.7)	111 (41.3)	189 (43.3)	431 (51.4)	
Years of education					
> 9 years	205 (37.0)	31 (11.5)	48 (11.0)	71 (8.5)	<0.001
≤ 9 years	349 (63.0)	238 (88.5)	389 (89.0)	767 (91.5)	
Smoking status					
Never/former smoker	491 (88.6)	251 (93.3)	393 (89.9)	699 (83.4)	<0.001
Current	46 (8.3)	13 (4.8)	29 (6.6)	119 (14.2)	
Diabetes					
No	463 (83.6)	213 (79.2)	334 (76.4)	598 (71.4)	<0.001
Yes	91 (16.4)	56 (20.8)	103 (23.6)	240 (28.6)	

CPI: Community Periodontal Index; *Chi-squared test.

Table 2 shows the results obtained using the multinomial logistic regression models. Poor oral hygiene, a low level of education (≤ 9 years), and the presence of diabetes were statistically significantly associated with the presence of calculus: OR = 5.04 (3.63 – 7.00), p < 0.001; OR = 4.38 (3.05 – 6.25), p < 0.001, and OR = 1.53 (1.09 – 2.14), p = 0.012,respectively. Other indicators, such as age and gender, were associated with the presence of calculus. Similarly, age ≥ 65 years (OR = 1.33 [1.03 – 1.72], p = 0.025), poor oral hygiene (OR = 6.86 [5.08 – 9.26], p < 0.001), a low level of education (≤ 9 years) (OR = 4.84 [3.51 – 6.66], p < 0.001), smoking (OR = 1.51 [1.05 – 2.18], p = 0.025), and the presence of diabetes (OR = 1.73 [1.28 – 2.32]; p < 0.001) were statistically significantly associated with the presence of periodontal pockets ≥ 4 mm.

**Table 2:** Multinomial logistic regression model for the association between education level and CPI Score among Mexican adults aged ≥ 50 years (n = 2098)

Variables		CPI = 0	CPI = 1	CPI = 2	CPI = 3 or 4
			Odds Ratio (95%CI)	Odds Ratio (95%CI)	Odds Ratio (95%CI)
Gender	Male	Reference	Reference	Reference	Reference
	Female	Reference	0.93 (0.67 – 1.27) p = 0.665	0.64 (0.49 – 0.85) p = 0.002	0.98 (0.76 – 1.26) p = 0.902
Age	50–64 years	Reference	Reference	Reference	Reference
	≥ 65 years	Reference	1.01 (0.73 – 1.40) p = 0.929	0.70 (0.52 – 0.94) p = 0.020	1.33 (1.03 – 1.72) p = 0.025
Oral hygiene (OHI-S)	Good hygiene	Reference	Reference	Reference	Reference
	Poor hygiene	Reference	4.62 (3.22 – 6.63) p < 0.001	5.04 (3.63 – 7.00) p < 0.001	6.86 (5.08 – 9.26) p < 0.001
Years of education	> 9 years	Reference	Reference	Reference	Reference
	≤9 years	Reference	3.88 (2.54 – 5.93) p < 0.001	4.38 (3.05 – 6.25) p < 0.001	4.84 (3.51 – 6.66) p < 0.001
Smoking status	Never/former smoker	Reference	Reference	Reference	Reference
	Current	Reference	0.72 (0.42 – 1.23) p = 0.236	1.32 (0.88 – 1.99) p = 0.171	1.51 (1.05 – 2.18) p = 0.025
Diabetes	No	Reference	Reference	Reference	Reference
	Yes	Reference	1.18 (0.80 – 1.75) p = 0.382	1.53 (1.09 – 2.14) p = 0.012	1.73 (1.28 – 2.32) p < 0.001

OR: odds ratio; CI: confidence interval. Model adjusted for age, gender, oral hygiene (OHI-S), years of education, smoking status, diabetes.

## DISCUSSION

The results of the present study showed that adults age ≥ 50 years with a low level of education present worse periodontal health (periodontal pockets ≥ 4 mm in depth) than those adults with a higher level of education, after the regression model was fit for possible confounders. Level of education is one of the intermediary social determinants found to be related to the well-being of older adults. In general terms, an individual’s health improves with their level of education, because they develop habits, skills, and resources that enable them to improve their own health.^38^ According to the literature, individuals with a high level of education have a high level of personal control and simply more information. They know more about health, tending to adopt a healthy lifestyle and carry out preventive actions to take care of themselves.^38^ Even in developed countries, such as the United States, it has been observed that adults with a lower level of education present poorer health than other populations.^44^

Level of education plays a significant role in the processes affecting oral health. Yamamoto et al^43^ reported that patients with a low level of education have worse periodontal health and a lower number of teeth than those with a high level of education. Similarly, a meta-analysis conducted in this area of research found that a low level of education was associated with a higher risk of periodontitis in adults aged 35 years or over.^4^ The present study found that nearly 40% of adults ≥ 50 years of age presented periodontal pockets ≥ 4 mm in depth, with approximately 83.0% of this cohort having ≤ 9 years of education. Possible reasons why Mexican adults aged 50 years and over present more education-related oral health inequalities include, first and foremost, the treatment costs of dental visits, wherein it has been reported that older adults without education are 73% less likely (OR = 0.27; p < 0.001) to visit the dentist in the last year.^12^ Second, those with a lower level of education tend to have lower incomes and cannot afford dental treatment.^18^ Another possible explanation for the relationship found by the present study is the high levels of inequality in access to and use of oral healthcare services. Sánchez-García et al^29^ reported that approximately half of older Mexican adults with social security coverage have used oral healthcare services in the last 12 months. Therefore, the level of education in adults ages ≥ 50 years may affect their access to and use of oral healthcare services due the social inequalities they face. Oral healthcare strategies are required to assist in the diagnosis, prevention, and reduction of oral diseases, via self-care or simple evidence-based measures for the entire population, in order to significantly reduce the burden of disease and the negative impact on quality of life. It should be noted that education helps to promote and maintain healthy lifestyles and positive options, support personal relationships, and improve personal and family well-being, as well as that of the population.^27^

The present study found that poor oral hygiene increases the probability of bleeding, calculus, and periodontal pockets ≥ 4 mm in depth in adults ages 50 years or over. Plaque and the presence of calculus have been considered factors in the occurrence of gingivitis and its progression into periodontitis, which is mainly due to inadequate oral hygiene.^36,40^ Some studies have found that people with periodontitis present a higher percentage of calculus.^1,33^ The present study observed that 62.1% of adults sampled presented poor hygiene, an association that was significant for the CPI = 2 category (OR = 5.04; p < 0.001), meaning that poor oral hygiene generates a greater progression of the disease.^16^

The association between diabetes and severe periodontitis has been widely studied in different populations.^17,39^ The present study found that, in Mexican adults, the presence of diabetes was related to the presence of calculus and periodontal pockets ≥ 4 mm in depth. Pranckeviciene et al^26^ found an association between severe periodontitis and diabetes (OR = 1.83; p = 0.047). The association reported between diabetes and severe periodontitis results from the accumulation of plaque and calculus and a lack of toothbrushing, a lack of periodontal treatment, and a lack of glycemic control in the long-term.^30^

It is known that smoking is risk factor for severe periodontitis due to its vasoconstricting effect,^13^ an effect that results in a reduction of the efficacy of the defense mechanisms of the gums. Research has demonstrated that smokers are more likely than non-smokers to have bone loss, mobility, and tooth loss, as well as increased pocket probing depth.^14^ Other studies have reported an association between smoking and severe periodontitis (OR = 1.40; p = 0.028).^15,21^ The present study found an association between smoking and the presence of periodontal pockets ≥ 4 mm in depth (OR = 1.51; p = 0.025).

Finally, the present study observed that adults aged ≥ 65 years and over were more likely to present periodontal pockets ≥ 4 mm in depth (OR = 1.33; p = 0.025). Other studies have reported that periodontitis is common in people aged over 65 years.^11,34^ Therefore, a higher prevalence of periodontal disease in adults, combined with the increased proportion of older people in the global population, may have an impact on the need for healthcare and dental services in the coming years. Similarly, long-term planning is required for oral health services to meet the needs of the continually increasing older adult population.^34^

The present study has various limitations. First, the results were based on cross-sectional data, which did not allow causal inferences. Second, the data collected is not representative of the general population and could lead to the overestimation of the prevalence of oral diseases, thus minimising the prevalence of periodontal disease in the Mexican population. Third, while six sites on each tooth were examined when the periodontal pockets were probed, periodontal probing was not calibrated and the variations between dentists at the sentinel units were not evaluated. Fourth, selection bias could present due to the fact that the participants of the present study had to attend the dental service offered at each sentinel unit. On the other hand, the present research is one of the few studies to have examined the relationship between level of education and periodontal disease in populations aged 50 years or over.

## CONCLUSIONS

The present cross-sectional study showed that a low level of education is associated with a worse periodontal status in adults ≥ 50 years of age. Similarly, poor oral hygiene, smoking, diabetes, and being over 65 years old are factors related to periodontal status. These results suggest the importance of periodontal education from an early age onward, as well as the need for effective strategies and interventions for reducing oral health inequalities, in order to thus reduce the gaps in access to oral healthcare over the course of an older adult’s life as they age.
